# The First FISH-Confirmed Non-Canonical Telomeric Motif in Heteroptera: *Cimex lectularius* Linnaeus, 1758 and *C. hemipterus* (Fabricius, 1803) (Hemiptera, Cimicidae) Have a 10 bp Motif (TTAGGGATGG)*_n_*

**DOI:** 10.3390/genes15081026

**Published:** 2024-08-05

**Authors:** Desislava Stoianova, Snejana Grozeva, Natalia V. Golub, Boris A. Anokhin, Valentina G. Kuznetsova

**Affiliations:** 1 Institute of Biodiversity and Ecosystem Research, Bulgarian Academy of Sciences, 1000 Sofia, Bulgaria; sngrov@gmail.com; 2 Zoological Institute, Russian Academy of Sciences, 199034 St. Petersburg, Russia; natalia.golub@zin.ru (N.V.G.); boris.anokhin@zin.ru (B.A.A.)

**Keywords:** bed bugs, chromosomes, fluorescence in situ hybridization, Heteroptera, telomeres

## Abstract

Fluorescence in situ hybridization (FISH) with two different probes, the canonical insect telomeric sequence (TTAGG)*_n_* and the sequence (TTAGGGATGG)*_n_*, was performed on meiotic chromosomes of two members of the true bug family Cimicidae (Cimicomorpha), the common bed bug *Cimex lectularius* Linnaeus, 1758 and the tropical bed bug *C*. *hemipterus* (Fabricius, 1803), whose telomeric motifs were not known. In both species, there were no hybridization signals with the first probe, but strong signals at chromosomal ends were observed with the second probe, indicating the presence of a telomeric motif (TTAGGGATGG)*_n_*. This study represents the first FISH confirmation of the presence of a non-canonical telomeric motif not only for the infraorder Cimicomorpha but also for the suborder Heteroptera (Hemiptera) as a whole. The present finding is of key significance for unraveling the evolutionary shifts in the telomeric sequences in this suborder.

## 1. Introduction

Telomeres are protective complexes at the ends of eukaryotic linear chromosomes containing head-to-tail short tandem DNA repeats and associated proteins. Telomeres ensure chromosome integrity and replication by protecting chromosomal ends from breakage through a telomere-specific multi-protein structure called the telomeric cap [1,2,3,4,5]. Dysfunctional telomeres can cause genomic instability and cell cycle arrest. In most eukaryotes, telomere-specific reverse transcriptase (enzyme telomerase), which elongates terminal DNA arrays using a small region of its RNA subunit as a template, is involved in telomere DNA maintenance [6]. Comparative analysis of telomeres in different groups of eukaryotes has shown that certain telomeric repeats are characteristic of some high-ranking taxa: (TTTAGGG)*_n_* for plants, (TTAGGC)*_n_* for nematodes, (TTAGGG)*_n_* for vertebrates, including humans, and (TTAGG)*_n_* for arthropods, including insects [7,8]. It should be noted, however, that the telomeric motifs in the groups listed above are known for a relatively small number of representatives, and in every group, there is a certain number of deviations from the canonical telomeric sequences, increasing as the number of species studied increases [9,10,11,12,13,14].

Until recently, knowledge of the insect telomeres was based almost exclusively on data obtained by FISH and other hybridization techniques [10,15]. The use of these techniques has shown that the canonical “arthropod/insect” motif (TTAGG)*_n_* has in some cases been secondarily replaced by alternative motifs, e.g., (TCAGG)*_n_* and (TTGGG)*_n_* in at least three superfamilies of the large order Coleoptera [16,17,18,19] or (TTAGGG)*_n_* in ants of the genus *Myrmecia* Fabricius, 1804 (Hymenoptera) [20]. However, in most cases where canonical telomeric sequences have not been found, the question about the organization and exact nature of telomeres remains open. Hybridization techniques widely used to identify telomeric motifs have a number of drawbacks [12,13,14]. First of all, they cannot distinguish between telomeric and subtelomeric sequences [12]. In addition, they cannot identify unknown motifs, so to be successful in this case, it is necessary to use different hybridization probes in a random order, which is not productive. 

Due to rapid and significant advances in genome sequencing technologies, the genomes of many insect species have been sequenced, and information on telomeric sequences has expanded. More recently, the use of chromosome-level genome assemblies (telomere-to-telomere) taken from publicly available databases, literature sources, or self-generated sources has provided invaluable insights into the telomeres of insects. Although this approach also has several drawbacks (see, e.g., [14]), high-quality genome assemblies represent a valuable resource for further studies of telomeric sequences. Using this approach, numerous cases of major evolutionary shifts in the telomeric motifs of insects were identified, and their diversity was found to be much greater than previously thought [21]. A total of 220 species belonging to 182 genera and 53 families have been analyzed [12,13,14], including four species from three families of the true bug infraorder Pentatomomorpha, such as Pentatomidae, Coreidae, and Acanthosomatidae [14]. 

The currently available FISH data on Heteroptera indicate that this group is heterogeneous with respect to telomere structure. Of about 68 species studied with respect to telomere motifs [22,23,24,25,26,27,28,29,30,31,32,33], only 17 species showed the (TTAGG)*_n_* motif. These are all species of the families Belostomatidae [24,30] and Nepidae [29] from the basal infraorder Nepomorpha, and all species of the family Reduviidae from the more advanced infraorder Cimicomorpha [28,31,32] studied so far (but see below). FISH and SBH analyses performed in all other Cimicomorpha species as well as in all species from the related Cimicomorpha infraorder Penatatomomorpha showed the absence of the TTAGG telomeric repeat (reviewed in [10]; see also [26,27,31,33,34]). Moreover, Southern Blot experiments with several TTAGG-negative species, including *Cimex lectularius* Linnaeus, 1758 (Cimicidae) and *Nabis* sp. (Nabidae) from Cimicomorpha and *Oxycarenus lavaterae* (Fabricius, 1787) (Lygaeidae) from Pentatomomorpha, excluded six alternative telomerase-based repeats [23], such as TTTTGGGG, TTGGGG, TTAGGC, TAACC, TTAGGG, and TTTAGGG; therefore, the telomeric sequence(s) in these families remains unknown. Furthermore, a very recent FISH study of a member of the cimicomorphan family Miridae, *Nesidiocoris tenuis* (Reuter, 1895), excluded several other short DNA sequences, namely (TATGG)*_n_*, (TTGGG)*_n_*, and (TCAGG)*_n_*, as the telomeric repeat motifs at its chromosomes [33].

Recently, however, a number of non-canonical 10-bp motifs have been predicted based on the chromosome-level genome assemblies for members of three other families of Pentatomomorpha [14]: TTAGGGATGG and TTAGGGGTGG for *Aelia acuminate* Linnaeus, 1758 and *Rhaphigaster nebulosa* (Poda, 1761), respectively (Pentatomidae); TTAGGGATGG for *Gonocerus acuteangulatus* (Goeze, 1778) (Coreidae); and TTAGGGTGGT for *Acanthosoma haemorrhoidale* (Linnaeus, 1758) (Acanthosomatidae). The detection of the same TTAGGGATGG repeat motif in *A. acuminate* and *G. acuteangulatus* deserves special attention and allows us to propose two alternative hypotheses. On the one hand, it can be assumed that this repeat arose independently in the evolution of the distantly related Coreidae (Coreoidea) and Pentatomidae (Pentatomoidea) families, and, on the other hand, it may represent the ancestral state of this trait in the infraorder Pentatomomorpha.

To shed light on this question, we set out to investigate whether the TTAGGGATGG sequence forms telomeres in representatives of the sister to the Pentatomomorpha infraorder Cimicomorpha. For this purpose, we applied FISH with (TTAGGGATGG)*_n_* and (TTAGG)*_n_* probes to male meiotic chromosomes of two species of the genus *Cimex* Linnaeus, 1758 (Cimicidae), *C. lectularius* and *C. hemipterus* Fabricius, 1803, whose telomeric sequences have not yet been determined. 

## 2. Materials and Methods

### 2.1. Sampling

The adult males of *C. lectularius* and *C. hemipterus* used in this study were collected in 2023, the former from a private residence in Sofia, Bulgaria, and the latter in Voronezh, Russia. Specimens were fixed alive in a fixative consisting of 96% ethanol and glacial acetic acid (3:1) and stored in the fixative at 4 °C until further use.

### 2.2. Chromosome Preparations and Fluorescence In Situ Hybridization with Probes for Telomeric Repeats

The squash method of chromosome preparations and the FISH procedure were performed as described previously [23,31]. For FISH, two DNA sequences labeled with dUTP-biotin by PCR, using the GGTTAGGGATGGTTAGGGATGG/TAACCATCCCTAACCATCCTAA and TAACCTAACCTAACCTAACCTAA/GGTTAGGTTAGGTTAGGTTAGG primers, without a DNA template, were used as probes: (TTAGGGATGG)*_n_* and, for control, the insect canonical telomeric sequence (TTAGG)*_n_*. 

Chromosome preparations were prepared in the following manner: they were dehydrated through 70/80/96% Ethanol at RT and treated with 100 μg/mL RNaseA (Sigma, Burlington, MA, USA) for 60 min at 37 °C in a humid chamber; washed three times in 2xSSC (5 min each) at RT; dehydrated through 70/80/96% Ethanol at RT; incubated in 5 mg/mL Pepsin in 0.01N HCl for 15 min at 37 °C; washed sequentially in 1xPBS, in PBSx1/0.05M MgCl_2_ for 5 min each and in 1% PFA in PBSx1/0.05M MgCl_2_ for 10 min, in 1x PBS for 5 min, and in PBSx1/0.05M MgCl_2_ for 5 min at RT each; dehydrated through 70/80/96% Ethanol at RT or ice cold; and, finally, dried. After pretreatment, a hybridization mixture containing 50–100 ng of labeled probe, 50% formamide, 2xSSC, 10% (*w*/*v*) dextran sulfate, 1% (*w*/*v*) Tween-20, and 10 µg salmon-sperm DNA was added to the preparations. Slides were mounted using glass coverslips and rubber cement. The slides were denaturated for 5 min at 75 °C. Then the chromosome slides were incubated for 42–44 h at 37 °C. Following hybridization, the slides were washed first in 2xSSC for 3 min at 45 °C, then in 50% formamide in 2xSSC for 10 min at 45 °C, and then two more times in 2xSSC (10 min each) at 45 °C; they were then blocked in 1.5% (*w*/*v*) BSA/4xSSC/0.1% Tween-20 for 30 min at 37 °C in a humid chamber. The probes were detected with 5 μg/mL Avidin-Alexa Fluor 488 (Invitrogen, Waltham, MA, USA). Detection reaction was performed in 1.5% BSA/4xSSC/0.1% Tween-20 for 1 h at 45 °C. The slides were washed three times in 4xSSC/0.02% Tween-20 (10 min each) at 45 °C and dehydrated through 70/80/96% ethanol at RT. Chromosomes were mounted in a mounting-antifade (ProLong Gold antifade reagent with DAPI, Invitrogen, Waltham, MA, USA) and covered with a glass coverslip.

### 2.3. Microscopy

FISH preparations were analyzed under a Leica DM 6000 B microscope, and images were acquired using a Leica DFC 345 FX camera and Leica Application Suite 3.7 software with the Image Overlay module.

The specimens from which the chromosome preparations were obtained, as well as the preparations themselves, are stored at the Zoological Institute of the Russian Academy of Sciences (St. Petersburg, Russia) and the Institute of Biodiversity and Ecosystem Research of the Bulgarian Academy of Sciences (Sofia, Bulgaria).

## 3. Results and Discussion

The two species we studied belong to the genus *Cimex* of the true bug family Cimicidae (Cimicomorpha). These are specialized hematophagous ectoparasitic insects, grouped into 24 genera, with about 100 species worldwide [35]. *Cimex* is one of the most recognized and discussed genera of Cimicomorpha. It represents a small group of insects known as temporary but obligatory ectoparasites of humans, bats, and birds [36,37]. Although taxonomy is still debated, the genus includes about 23 currently recognized species [38]. The two target species, commonly referred to as bed bugs (species that feed predominantly on human blood), are the common bed bug, *C. lectularius*, distributed mainly in temperate zones, and the tropical bed bug, *C. hemipterus*, more commonly found in tropical and subtropical areas [39].

**Figure 1:**
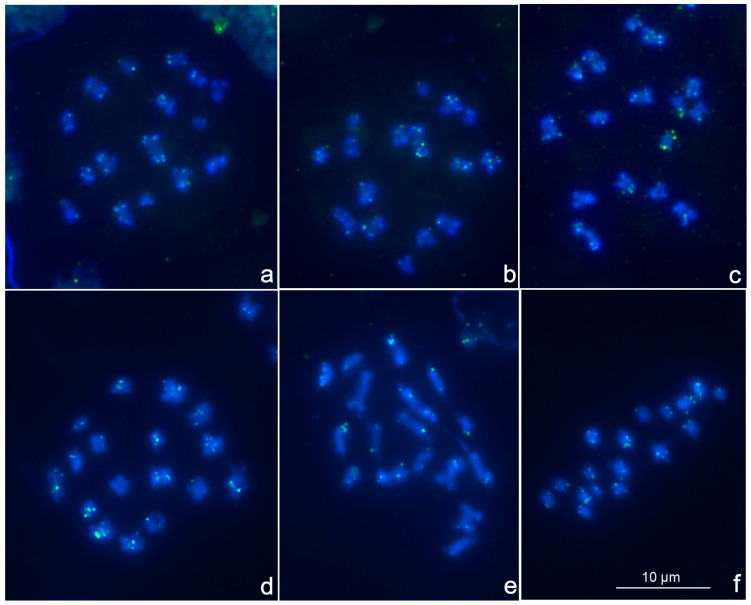
Male metaphase I plates after FISH with (TTAGGGATGG)***_n_*** probe: (**a**–**c**)—*C. lectularius*, n = 13 + X_1_X_2_Y (2n = 26 + X_1_X_2_Y); (**d**–**f**)—*C. hemipterus*, n = 14 + X_1_X_2_Y (2n = 28 + X_1_X_2_Y).

We performed FISH with probes against (TTAGG)*_n_* and (TTAGGGATGG)*_n_* telomere repeat sequences on chromosome preparations of both species. We showed that male meiotic karyotypes of *C. lectularius* and *C. hemipterus* include n = 16(13 + X_1_X_2_Y) and 17(14 + X_1_X_2_Y), respectively (Figure 1), as previously reported by other researchers [23,40,41]. It should be noted that the first and so far only chromosome-level genome assembly for the family Cimicidae, just obtained by Low et al. [42] for *C. hemipterus*, contained 16 pseudochromosomes (the Y chromosome was not assembled into the genome). The telomeric sequences of the species were not commented on in the cited paper, because telomeres were not assembled in the presented pseudochromosome assembly (we reached this conclusion after examining the available online assembly with access number: JASJUQ000000000 in NCBI accessed on 10 June 2024).

As mentioned above, *C. lectularius* lacks the canonical insect telomeric motif (TTAGG)*_n_* [23], whereas nothing similar was previously known about *C. hemipterus*. In our study, FISH with the 5-bp probe (TTAGG)*_n_* did not yield hybridization signals in either *C. lectularius* or *C. hemipterus*, as expected, whereas FISH with the 10-bp probe (TTAGGGATGG)*_n_* yielded distinct hybridization signals at chromosome ends in both species, indicating the presence of a telomere motif (TTAGGGATGG)*_n_* (Figure 1).

Studies of insect telomeres have shown that telomeric repeat motifs are usually (although there are exceptions, as discussed above) conserved in high-level taxa [10,15,19]. Until recently, this seemed to be true also for Heteroptera, in which, for example, all species of the family Reduviidae (Cimicomorpha) studied so far have the (TTAGG)*_n_* motif [28,31,32]. However, the finding of three distinct motifs in the Coreidae, Acanthosomatidae, and Pentatomidae families, with two motifs in the Pentatomidae family, shows that this is not the case. 

**Figure 2:**
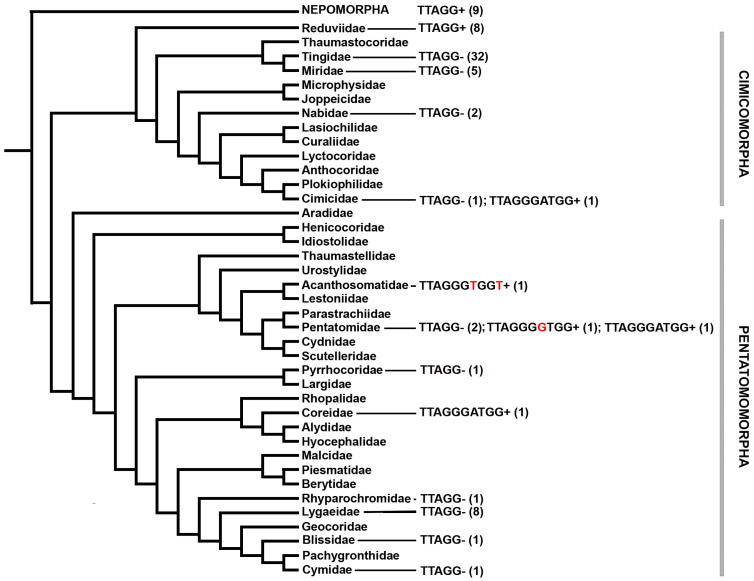
Telomeric repeats found by FISH or predicted from whole-genome data in various families of Cimicomorpha, Pentatomomorpha, and (as an outgroup) Nepomorpha, mapped on the true bug phylogeny (after Weirauch et al. [43]).

The detection of the (TTAGGGATGG)*_n_* motif in very distant groups, such as the Coreidae and Pentatomidae families, on the one hand, and in the family Cimicidae, on the other, suggests that this motif may be more common in the sister phylogenetic lineages Pentatomomorpha and Cimicomorpha. Available data on telomeric repeats found by FISH or predicted from chromosome-level genome assemblies in different families of Cimicomorpha, Pentatomomorpha, and (as an outgroup) Nepomorpha are presented in Figure 2 (the number of species studied for a particular telomeric motif is given in parentheses). Data on the presence of a particular repeat (and the absence of the insect-type TTAGG repeat) in the families were obtained from the following sources: Reduviidae [28,31,32]; Tingidae [25,26]; Miridae [23,33]; Lygaeidae, including two other lygaeioid families—Heterogastridae and Oxycarenidae [23,27,34]; Pyrrhocoridae [22,23]; Nabidae [31]; Cimicidae [23] and the present study; Pentatomidae [14,23]; Coreidae and Acanthosomatidae [14]; and Nepomorpha [24,29,30].

## 4. Conclusions

We have shown that the telomeres of *C. lectularius* and *C. hemipterus* consist of the (TTAGGGATGG)*_n_* motif. This is the first FISH-confirmed presence of a non-canonical telomeric repeat for the infraorder Cimicomorpha and for the suborder Heteroptera in general. It seems clear that the combined use of chromosome-level genomic assemblies and FISH promises a breakthrough in understanding the diversity of telomeric sequences in the suborder Heteroptera, in which most species studied lack the insect-type TTAGG telomeric repeat. 

## Data Availability

Data are contained within the article.
